# A novel randomly textured phosphor structure for highly efficient white light-emitting diodes

**DOI:** 10.1186/1556-276X-7-188

**Published:** 2012-03-16

**Authors:** Hsin Chu Chen, Kuo Ju Chen, Chao Hsun Wang, Chien Chung Lin, Chia Chi Yeh, Hsin Han Tsai, Min Hsiung Shih, Hao Chung Kuo, Tien Chang Lu

**Affiliations:** 1 Department of Photonics and Institute of Electro-Optical Engineering, National Chiao Tung University, 1001 University Road, Hsinchu, 300, Taiwan; 2 Institute of Photonic System, National Chiao Tung University, 301 Gaofa 3rd Rd., Guiren Township, Tainan County, 711, Taiwan; 3 Research Center for Applied Sciences, Academia Sinica 128 Academia Rd., Sec 2 Nankang, Taipei, 115, Taiwan

**Keywords:** white light-emitting diodes, imprinting, phosphor, texture, package

## Abstract

We have successfully demonstrated the enhanced luminous flux and lumen efficiency in white light-emitting diodes by the randomly textured phosphor structure. The textured phosphor structure was fabricated by a simple imprinting technique, which does not need an expensive dry-etching machine or a complex patterned definition. The textured phosphor structure increases luminous flux by 5.4% and 2.5% at a driving current of 120 mA, compared with the flat phosphor and half-spherical lens structures, respectively. The increment was due to the scattering of textured surface and also the phosphor particles, leading to the enhancement of utilization efficiency of blue light. Furthermore, the textured phosphor structure has a larger view angle at the full width at half maximum (87°) than the reference LEDs.

## Introduction

In recent years, white light-emitting diodes (LEDs) have become important sources of illumination because of their high brightness, reliability, low power consumption, and long lifetime compared to conventional lighting sources [1]. By far, white LED composed of GaN-based chip and yellow phosphor is the most promising and efficient method [2]. The principle of phosphor-converted white LEDs was employing a short-wavelength LED to excite the wavelength conversion phosphor and down-convert high-energy photons to longer wavelength ones, and the combined photons create a perceived white spectrum [3]. The advantages of this method are low cost, simple fabrication, and high conversion efficiency, comparing to individual red, green, and blue LEDs mixing and UV-LEDs exciting red, green, and blue phosphors [4].

However, for high-power application, there are still some imperfections left to be optimized and improved, such as the limited extracted power due to lead-frame package, silicone encapsulant, and the backscattered light. The backscattered light is not only unusable but also leads to reliability issues. Therefore, some methods such as scattered photon extraction package [5], remote phosphor package [6-8], and ring remote phosphor structure [9] are developed to better utilize the emitted photons and the design of optical lens. Due to a large difference in the refractive index between GaN and air, most of the light emission is trapped internally in LEDs. Therefore, the roughening or nano-texturing of surface was frequently adopted in GaN-based blue LEDs to increase extraction efficiency [10-12]. This method can also excellently increase the amount of blue light to excite the yellow phosphor in white LEDs, but the blue and yellow light still experience total internal reflection at the interface of air and silicone glue, which leads to the limitation of lumen efficiency in white LEDs. On the other hand, high light extraction efficiency could be attained by the design with a half-spherical lens, but the cost of the mold machine is high, and application is limited due to the large volume of the lens.

In this work, we further improved the luminous flux and lumen efficiency of white LEDs by a simple imprinting technique to form the textured phosphor structure, denoted as LED III. The LEDs with flat phosphor and half-spherical lens structures were denoted as LEDs I and II. With this textured surface, the total internal reflection at the interface of air and silicone could be reduced. Furthermore, this surface increases the probability of blue light scattering, which is supported by haze measurement results. Accordingly, more blue light can excite phosphor again due to the enhancement of utilization efficiency of blue light. Meanwhile, more yellow light can be extracted by the textured phosphor structure. Additionally, we further investigate the far-field emission pattern for LED I, LED II, and LED III, respectively.

## Experimental process

**Figure 1:**
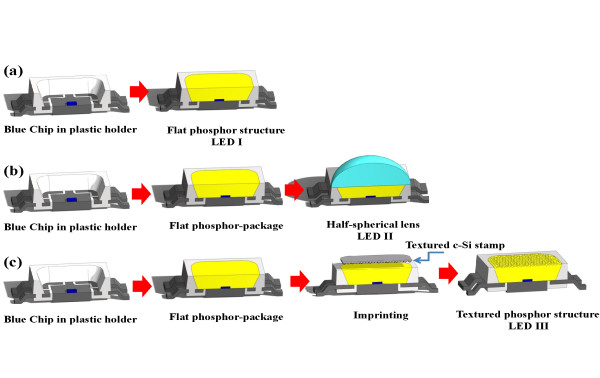
**Process flow charts of flat phosphor**. (a) without half-spherical lens (b) with half-spherical lens, and (c) textured phosphor of packages.

**Figure 2:**
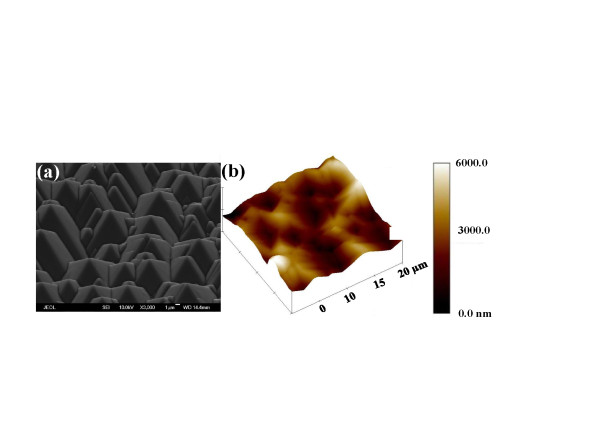
**SEM and AFM measurements**. **(a**) SEM image of randomly textured c-Si mold pattern and (**b**) AFM image of the textured phosphor structure.

In this experiment, we use the same blue LED chips and the same amount of phosphor mixed with silicone encapsulant for LEDs I, II, and III. A simple imprinting technique was applied to fabricate the textured phosphor structure in LED III. The process flow charts are illustrated in Figure 1. First, the GaN-based vertical-injection LEDs with a chip size of 24 mil^2 ^and emission wavelength of about 450 nm were placed in the commercial plastic lead-frame package by silver paste and wire-bonding. Second, the phosphor used in this experiment was Y_3_Al_5_O_12 _(YAG) phosphor. The phosphor was uniformly mixed with the silicone and then filled in the lead-frame by dispensing technique; it was denoted as LED I and shown in Figure 1, row a. Third, we put the half-spherical lens onto the reference LED I by the lens molding machine; it was denoted as LED II and shown in Figure 1, row b. Fourth, a randomly textured crystalline silicon (c-Si) mold pattern was used for imprinting by wet-etching with potassium hydroxide (KOH) [13,14]. Meanwhile, Figure 2a shows the scanning electron microscope (SEM) image of the c-Si substrate after randomly textured etching. The height of the randomly textured structure was about 4.2 μm. Next, a solution of de-molding on the surface of the c-Si mold pattern was fabricated by a spin-coating technique and then phosphor imprinting to form an inverted textured phosphor structure on the surface of the package; this was denoted as LED III and shown in Figure 1, row c. The atomic force microscopy (AFM) measurement of the textured phosphor surface shows the RMS of 1.2 μm, as shown in Figure 2b, indicating that the randomly textured c-Si pattern was successfully transferred onto the top of the phosphor layer.

## Results and discussion

**Figure 3:**
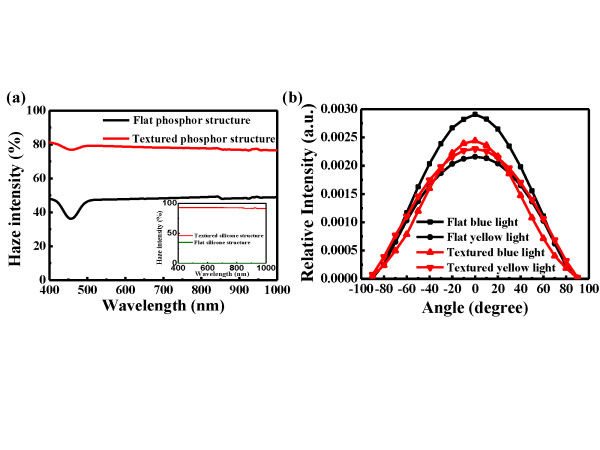
**Wavelength-dependent haze intensity and angular-dependent relative intensity measurements**. (**a**) The measured wavelength-dependent haze intensity with phosphor in flat/textured silicone structures; the inset is the flat/textured silicone structures without phosphor, and (**b**) measured angular-dependent relative intensity of the flat and textured phosphor structures at a driving current of 120 mA.

To understand the light scattering capability of the flat and textured phosphor structures in the visible light range, we measured the total transmittance and the diffractive transmittance by ultraviolet-visible spectrophotometry. By using the above measured results, the flat and textured phosphor structures of haze intensity can be calculated as follows:

(1) $$\text{Haze}\;\text{intensity}=T_{\text{diffraction}}/)T_{\text{total}}\times 100\%$$

(2) $$\text{Absorption}=100\%-T_{\text{total}}-R_{\text{total}}$$

where *T*_diffraction _was the measurement of the diffractive transmittance (not including zero order), *T*_total _was the measurement of the total transmittance, and *R*_total _was the measurement of the total reflectance. The inset of Figure 3a shows the wavelength-dependent haze intensity of the flat and textured silicone structures (no phosphor in the silicone). The large differences of haze intensity between the textured silicone (92%) and the flat silicone (0%) structures are presented. This result indicates that the textured silicone provides a refractive index gradient and facilitates the escape of photons from the silicone layer. Furthermore, we also measured the haze intensity of the textured phosphor and flat phosphor structures, as shown in Figure 3a. It was noticeable that the haze intensity of the textured phosphor structure can reach 80%, and the flat phosphor structure shows only 45%. Besides, the dip at the wavelength range between 420 and 500 nm was the absorption of the YAG phosphor. By the haze intensity measurement, we found that part of the light scattering is coming from the phosphor particles. Therefore, the exact absorption of the scattering light should be characterized in more details shown in the next section.

According to the above equation (Equation 2), the total absorption of the flat and textured phosphor structures can be calculated, which were 24% and 30% at the wavelength of 450 nm. The total enhancement of absorption can reach 25%, which leads to more blue light trapped in the textured phosphor structure. Hence, more yellow light could be produced due to this recycling of photons. Therefore, we further measured the angular-dependent relative intensity of LED I and LED III at a driving current of 120 mA, as shown in Figure 3b. It was clear that the intensity of blue light in the textured phosphor structure is lower than in the flat phosphor structure, which could be attributed to the increased blue light scattering and re-absorption. The result indicates the enhancement of utilization efficiency in blue light by the textured phosphor structure, and more yellow light can be extracted. Therefore, LED III has the higher relative intensity of yellow light than LED I.

**Figure 4:**
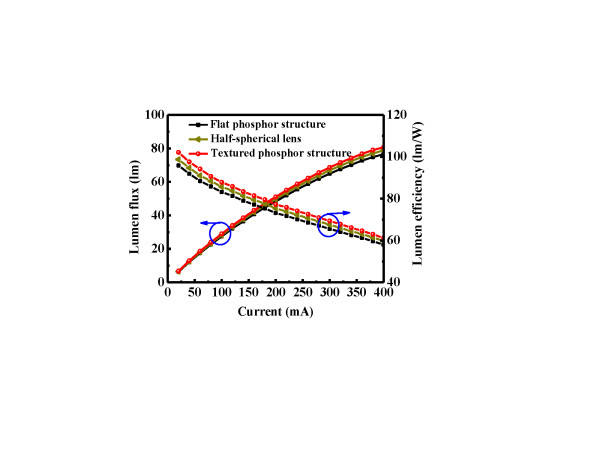
**Lumen efficiency and luminous flux results**. Current-dependent lumen efficiency and luminous flux of flat phosphor, half-spherical lens phosphor, and textured phosphor structures.

Figure 4 shows the current-dependent lumen efficiency and luminous flux of LEDs I, II, and III. The output power of bare GaN-based blue LED was about 100 mW at 120 mA. The luminous flux of LEDs I, II, and III were 32.2, 33.16, and 33.97 lm, respectively. By texturing the phosphor structure, the enhancement of luminous flux was about 5.4% higher than that of the flat phosphor structure, and these two LEDs' correlated color temperature was 4,324 K and 4,644 K, respectively. The reason for the increment was the enhancement of utilization efficiency in blue light, so the enhancement of yellow light was absorbed and extracted by the textured phosphor structure. Simultaneously, the higher probability of photon may have escaped from a refractive index gradient of the phosphor layer. Moreover, LED II with half-spherical lens structures shows only 3% of enhancement in luminous flux, which was lower than LED III.

**Figure 5:**
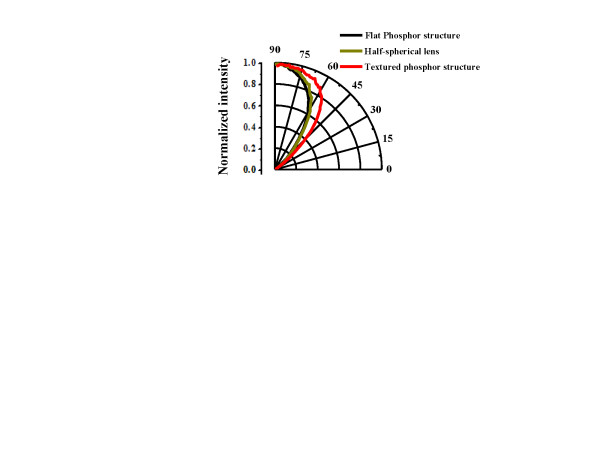
**Far-field emission measurement**. Measurement of far-field emission pattern of flat phosphor, half-spherical lens phosphor, and textured phosphor structures.

In order to confirm whether the enhancement of light scattering of the textured phosphor structure will affect the change of far-field emission pattern, we further measured the far-field emission of LEDs I, II, and III, as shown in Figure 5. The flat phosphor and half-spherical lens structures package shows almost the same view angle at the full width at half maximum (68° and 70°). However, the view angle of the textured phosphor package was enlarged to 87° because the total internal reflection is significantly suppressed by the textured phosphor structure. With the smaller package volume, brighter output, and larger view angle, the textured phosphor package shows more potential applications to conventional white LED package.

## Conclusion

In summary, we successfully proposed a simple method to use imprinting technique with a wet-etched c-Si substrate to fabricate the textured phosphor structure. The haze intensity of the textured phosphor structure shows 78% enhancement, compared with the flat phosphor structure. With the textured phosphor structure, the total internal reflection at the interface of air and silicone could be reduced. Furthermore, this surface increases the probability of blue light scattering, which is supported by the haze measurement results. Accordingly, utilization efficiency of blue light is greatly enhanced. Meanwhile, more yellow light can be extracted by the textured phosphor structure. As a result, the enhancement of the luminous flux was 5.4% and 2.5% higher than in the flat phosphor and lens molding packages at a driving current of 120 mA, respectively. The advantages of the textured phosphor structure are more compact package, higher lumen output, and larger view angle, which could provide flexible designs to various applications.

## Abbreviations

AFM: atomic force microscopy; c-Si: crystalline silicon: KOH: potassium hydroxide; LEDs: light-emitting diodes; LED I: flat phosphor package; LED II: lens molding package; LED III: textured phosphor package; SEM: scanning electron microscope; YAG: Y_3_Al_5_O_12_.

## Competing interests

The authors declare that they have no competing interests.

## Authors' contributions

HCC participated in the design of the study, measured the SEM and ultraviolet-visible spectrophotometer, explained the SEM image and haze intensity, and contributed in the writing of the manuscript. KJC participated in the discussion of the study, measured the optical characteristic, and explained luminous flux and lumen efficiency. CHW participated in the discussion of the study, measured the far-field emission, and explained it. CCY fabricated all the samples and measured the AFM. HHT fabricated all the samples and measured the luminous flux and lumen efficiency. CCL participated in the revision of the manuscript and discussion of the results. MHS participated in the discussion of the study. TCL participated in the discussion of the study. HCK participated in the discussion of all the results. All authors read and approved the final manuscript.
